# High expression of ACE2 and TMPRSS2 and clinical characteristics of COVID-19 in colorectal cancer patients

**DOI:** 10.1038/s41698-020-00139-y

**Published:** 2021-01-21

**Authors:** Chao Liu, Kai Wang, Min Zhang, Xiaoyu Hu, Tian Hu, Yumei Liu, Qinyong Hu, Shikai Wu, Jinbo Yue

**Affiliations:** 1 Department of Oncology, Renmin Hospital of Wuhan University, Wuhan, China; 2 Department of Radiation Oncology, Shandong Cancer Hospital and Institute, Shandong First Medical University and Shandong Academy of Medical Sciences, Jinan, China; 3 Department of Critical Care Medicine, Zhujiang Hospital, Southern Medical University, Guangzhou, China; 4 The Fifth Medical Center of Chinese PLA General Hospital, Beijing, China; 5 Department of Respiratory Medicine, Wuhan Hankou Hospital, Wuhan, China; 6 Department of Medical Oncology, Peking University First Hospital, Beijing, China

**Keywords:** Cancer, Cancer

## Abstract

Little is known of the patterns of expression of ACE2 and TMPRSS2 or the clinical characteristics of COVID-19 in patients with COVID-19 and colorectal cancer. We found in both bulk and single-cell RNA-seq profiles that ACE2 and TMPRSS2 were expressed at high levels on tumor and normal colorectal epithelial tissues. Clinically, patients with colorectal cancer and COVID-19 were more likely to have lymphopenia, higher respiratory rate, and high hypersensitive C-reactive protein levels than matched patients with COVID-19 but without cancer. These results suggest that patients with colorectal cancer may be particularly susceptible to SARS-CoV-2 infection. Further mechanistic studies are needed to support our findings.

In late 2019, a new RNA coronavirus, severe acute respiratory syndrome coronavirus 2 (SARS-Cov-2), was found to have infected humans, and led to a worldwide outbreak of coronavirus disease (COVID-19)^1,2^. By 4 October 2020, more than 35 million confirmed cases had been reported across 214 countries, areas, or territories, resulting in more than 1,000,000 deaths.

Previous studies have shown that COVID-19 patients often experience gastrointestinal symptoms such as diarrhea, anorexia, nausea, and vomiting^3–10^. SARS-CoV-2 viral RNA has been found to be present in fecal samples and to remain there longer than in respiratory samples^8,10,11^. Enterocytes in cultured human small intestinal organoids were shown by confocal and electron microscopy to have been infected by SARS-CoV-2^12^. SARS-CoV-2 can enter human cells through two entry receptors, angiotensin I-converting enzyme 2 (ACE2) and transmembrane serine protease 2 (TMPRSS2), and infection with the virus can lead to severe adverse outcomes such as acute respiratory distress and gastrointestinal syndromes^13^. Systematic investigation of the distribution of the ACE2 and TMPRSS2 in human tissues may benefit our understanding of the pathogenesis of SARS-CoV-2 infection. Several studies have shown ACE2 receptors to be expressed at high levels in the intestinal epithelium, which may explain the ability of the virus to infect the intestinal epithelium^4,12^. Cancer patients are known to be vulnerable to infection in general and may experience more serious consequences of COVID-19^14–17^, but little is known of whether ACE2 and TMPRSS2 are expressed in colorectal cancer tissues, or how infection with SARS-CoV-2 may affect the clinical course of patients with colorectal cancer.

In this study, we measured the expression of ACE2 and TMPRSS2 in colorectal cancer tissue samples from two publicly available databases by using bulk and single-cell RNA-sequencing (scRNA-seq). Our ultimate goal was to investigate the potential susceptibility of patients with colorectal cancer to infection with SARS-CoV-2. To further investigate the potential consequences of COVID-19 for patients with colorectal cancer, we also compared clinical characteristics, laboratory findings, and outcomes of five patients with colorectal cancer and COVID-19 with those of 20 matched patients with COVID-19 but without cancer.

## Epithelial cells in colorectal tumors and normal tissues express high levels of ACE2 and TMPRSS2 RNA

**Fig. 1:**
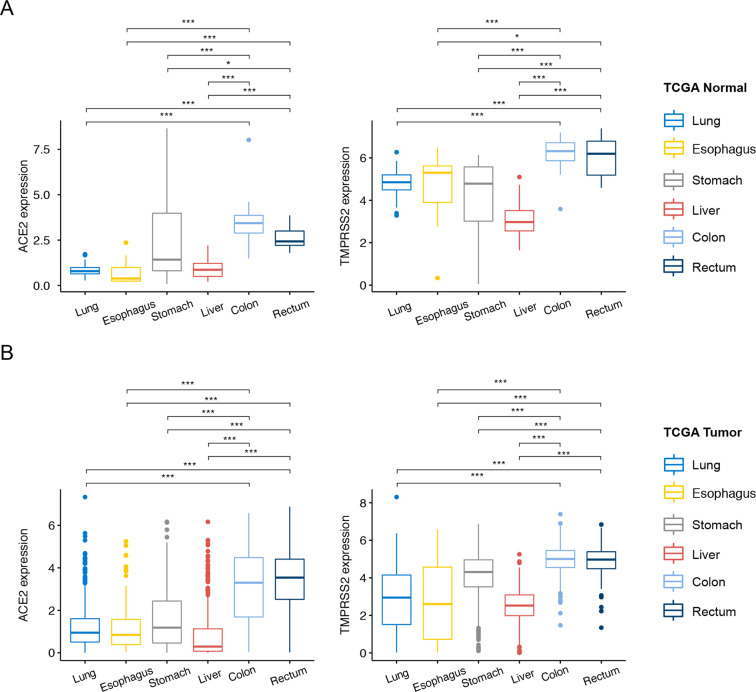
ACE2 and TMPRSS2 expression in normal and tumor tissues. **A** Box plots of ACE2 and TMPRSS2 expression in human normal tissue samples of colon, rectum, lung, esophagus, stomach, and liver from The Cancer Genome Atlas (TCGA). **B** Box plots of ACE2 and TMPRSS2 expression in human tumor tissues of colon, rectum, lung, esophagus, stomach, and liver from TCGA. Centre line, bounds of box and whiskers represent median value, quartile, and the most extreme data point that is no more than 1.5 × interquartile range beyond the box, respectively. Asterisks indicate significant differences (i.e., *P* < 0.05).

On the basis of previous findings that ACE2 and TMPRSS2 RNA was expressed in human normal and cancerous lung tissues^18^, we attempted to extend these results by examining the expression of ACE2 and TMPRSS2 in human colorectal tumor and normal tissue samples in The Cancer Genome Atlas. Bulk RNA-sequencing profiling showed that ACE2 and TMPRSS2 were expressed at higher levels in human colorectal tumor and normal tissue samples than in human tumor or normal tissue samples of lung, esophagus, stomach, and liver (*P* for all <0.05, Fig. 1), indicating that the colorectum may be a likely route of infection with SARS-CoV-2 in addition to the lungs.

**Fig. 2:**
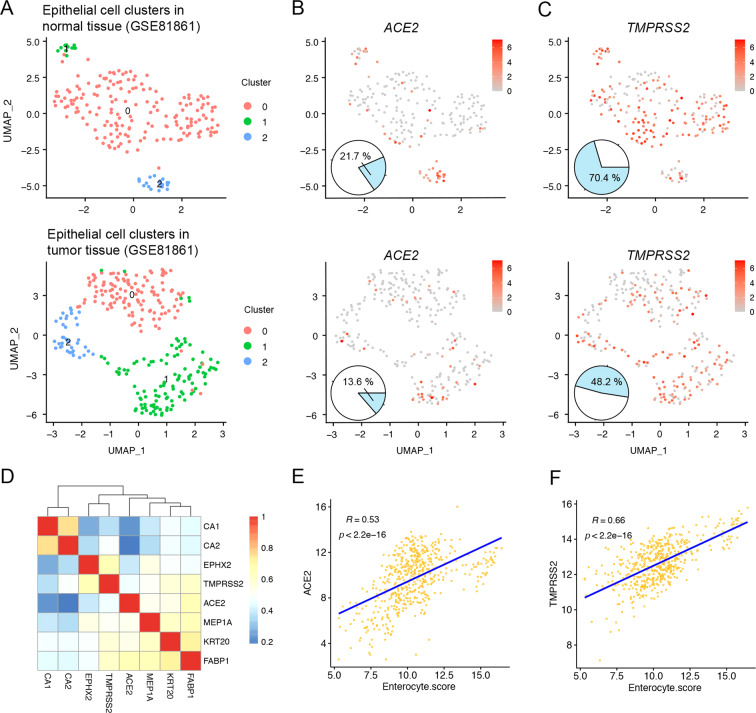
scRNA-seq of epithelial cells from human normal and tumor colorectal tissues. **A** Uniform manifold approximation and projection (UMAP) plot of epithelial cell clusters from human normal and tumor colorectal tissue samples. **B** UMAP and pie plots of ACE2 expression in epithelial cells from human normal and tumor colorectal tissue samples. **C** UMAP and pie plots of TMPRSS2 expression in epithelial cells from human normal and tumor colorectal tissue samples. **D** Heatmap shows correlation coefficients between ACE2 and TMPRSS2 and the indicated markers of enterocytes. **E**–**F** Scatter plots show correlation coefficients for ACE2 and TMPRSS2 with enterocyte scores.

**Fig. 3:**
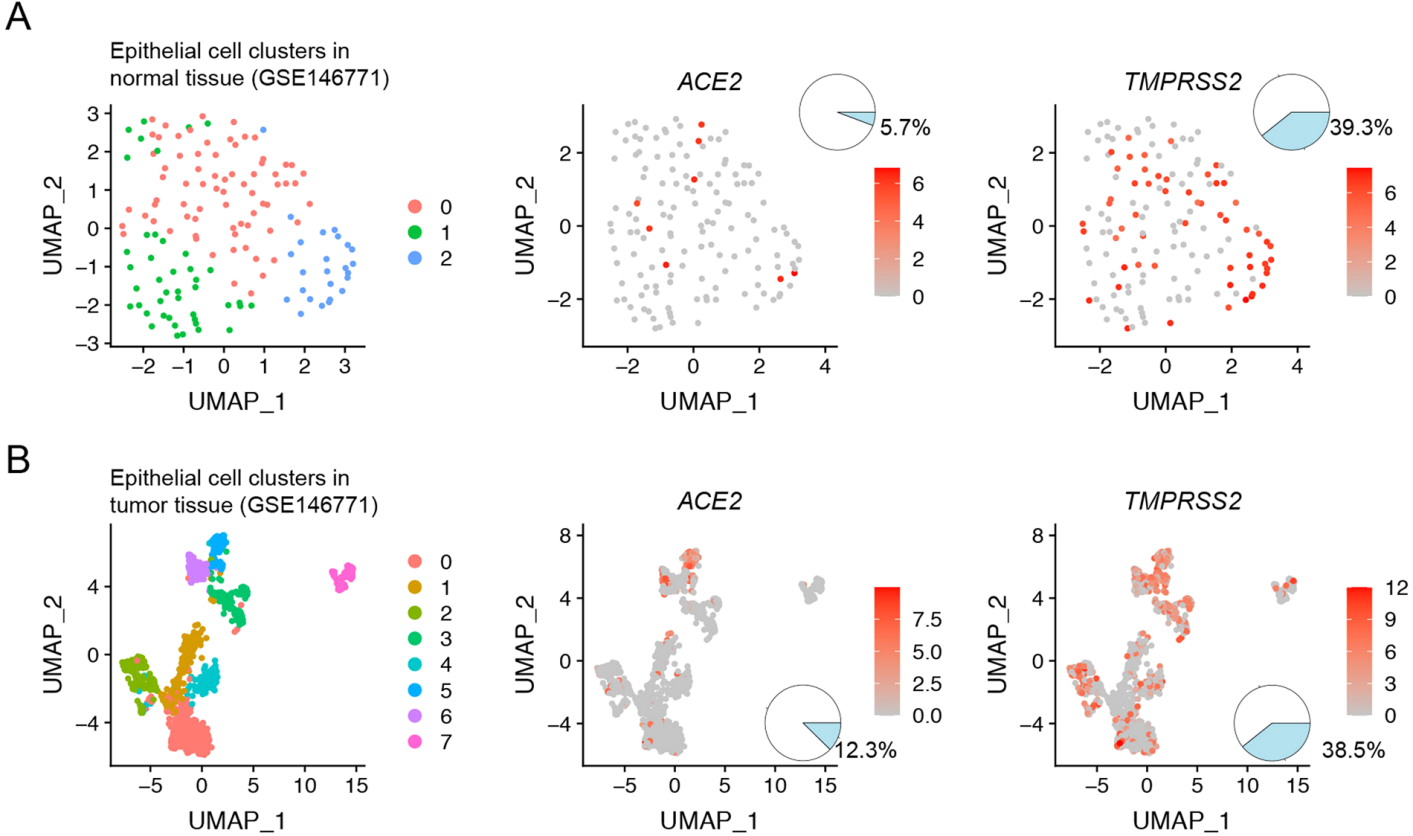
scRNA-seq of epithelial cells from human normal and tumor colon tissues. UMAP and pie plots of ACE2 and TMPRSS2 expression in epithelial cell clusters from human normal colon tissue samples (**A**) and from human tumor colon tissue samples (**B**).

We further analyzed the expression of ACE2 and TMPRSS2 in single-cell RNA-sequencing profiling of 11 pairs of colorectal tumor and colorectal normal tissues from 11 patients with colorectal cancer patients in the GSE81861 dataset. We found three clusters of epithelial cells in normal and tumor colorectal tissues (Fig. 2A). Among the 203 epithelial cells of 266 cells in normal colorectal tissues, 21.7% of cells expressed ACE2 and 70.4% expressed TMPRSS2; among the 272 epithelial cells of 375 cells in colorectal cancer tissues, 13.6% expressed ACE2 and 48.2% expressed TMPRSS2 (Fig. 2B–C). Given the possible link between ACE2 and TMPRSS2 distribution and susceptibility to SARS-Cov-2, we further performed Pearson correlation analysis of 6 additional markers of enterocytes (KRT20, CA1, CA2, EPHX2, MEP1A, FABP1) with ACE2 and TMPRSS2 in colorectal tissues. Expression of both ACE2 and TMPRSS2 was found to be highly correlated with the enterocyte score (Fig. 2D–F). We also analyzed single-cell RNA-sequencing profiles of pairs of colon tumors and normal colon tissue from 10 patients with colon cancer from the GSE146771 dataset. Among the 140 epithelial cells in normal colon tissues, 5.7% of cells expressed ACE2 and 39.3% expressed TMPRSS2; among the 989 epithelial cells in colon cancer tissues, 12.3% expressed ACE2 and 38.5% expressed TMPRSS2 (Fig. 3A–B). Collectively, these findings indicate that ACE2 and TMPRSS2 are expressed at high levels in both normal and cancerous colorectal tissues, suggesting that the colorectum may be particularly susceptible to SARS-CoV-2 infection.

## ACE2 and TMPRSS2 expression in non-epithelial cells from human normal and tumor colorectal tissues

**Fig. 4:**
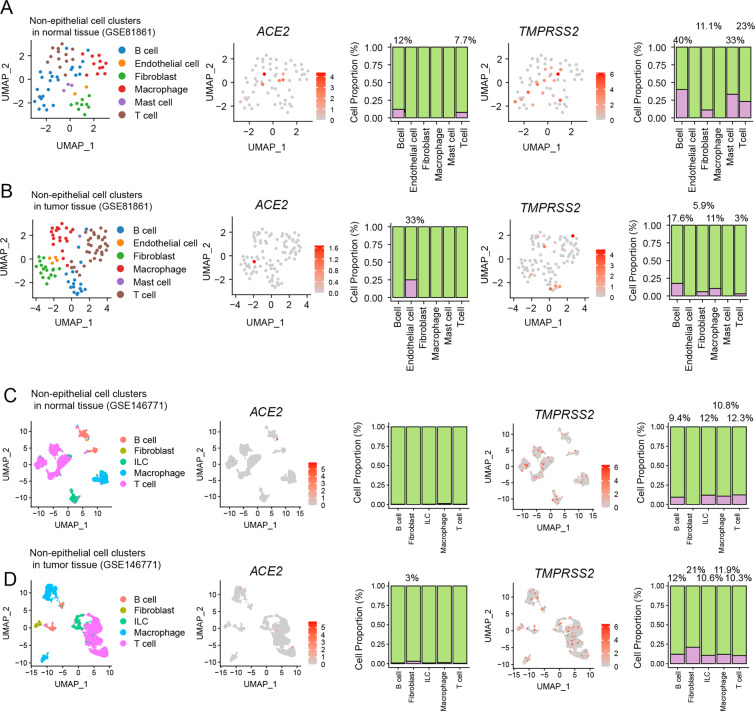
scRNA-seq of non-epithelial cells in human normal and tumor colorectal tissues. **A**–**B** UMAP and pie plots of ACE2 and TMPRSS2 expression in non-epithelial cell clusters from human normal tissues (**A**) and tumor colorectal tissue samples (**B**) from dataset GSE81861. **C**–**D** UMAP and pie plots of ACE2 and TMPRSS2 expression in non-epithelial cell clusters from human normal tissues (**C**) and tumor colon tissue samples (**D**) from GSE146771.

We also analyzed the expression of ACE2 and TMPRSS2 in non-epithelial cells (i.e., immune cells, endothelial cells, and fibroblasts) from human normal and tumor colorectal tissues. In the GSE81861 dataset, among 63 non-epithelial cells from human normal colorectal tissues, ACE2 was expressed by 12% of B cells and 7.7% of T cells; TMPRSS2 was expressed by 40% of B cells, 11.1% of fibroblasts, 33% of mast cells, and 23% of T cells (Fig. 4A). Also, in the GSE81861 dataset, among 103 non-epithelial cells from human colorectal cancer tissues, ACE2 was expressed by 33% of endothelial cells; TMPRSS2 was expressed by 7.6% of B cells, 5.9% of fibroblasts, 11% of macrophages, and 3% of T cells (Fig. 4B). In another dataset (GSE146771), among 5108 non-epithelial cells from human normal colon tissues, TMPRSS2 was expressed by 9.4% of B cells, 12% of innate lymphoid cells, 10.8% of macrophages, and 12.3% of T cells (Fig. 4C). Also, in GSE146771, among 4231 non-epithelial cells from human colon cancer tissues, ACE2 was expressed by 3% of fibroblasts; TMPRSS2 was expressed by 12% of B cells, 21% of fibroblasts, 10.6% of innate lymphoid cells, 11.9% of macrophages, and 10.3% of T cells (Fig. 4D).

## Severity of COVID-19 in patients with colorectal cancer and matched patients with COVID-19 without cancer

Cancer patients are thought to be more vulnerable to severe adverse effects from SARS-CoV-2 than are patients without cancer^14–17^, but whether this is also true for patients with colorectal cancer is unclear. To address this gap, we compared aspects of the severity of COVID-19 between patients with colorectal cancer and COVID-19 and matched patients with COVID-19 but without cancer. In this analysis, which was approved by the Ethical Committee of Wuhan Hankou Hospital, we assessed the clinical characteristics of 5 patients with colorectal cancer and COVID-19 admitted to Wuhan Hankou Hospital. COVID-19 was diagnosed on the basis of real-time polymerase chain reaction tests for SARS-CoV-2. The clinical characteristics of the 5 patients with COVID-19 and colorectal cancer, and those of 20 patients with COVID-19 without cancer, are shown in Table 1. Among the 5 patients with colorectal cancer, 2 (40%) were female and 3 (60%) were more than 65 years old; the most common comorbid condition was hypertension (60%), which was consistent with previous findings that COVID-19 patients often had hypertension^19,20^. All five patients had high hypersensitive C-reactive protein levels (i.e., >6 mg/L); 4 (80%) had lymphopenia (<0.8 × 10^9^/L), 4 (80%) had high procalcitonin levels (>0.05 ng/mL), and three had high lactate dehydrogenase levels (>300 U/L) and high D-dimer levels (>0.5 μg/mL); two patients had low total protein and three had low albumin levels. In terms of pulmonary function, four patients (80%) had high respiratory rates (>20/min) and 2 (40%) had low oxygen saturation levels (SpO_2_ < 93%). All patients presented with bilateral shadows on CT scans and fever, 4 (80%) had cough, 3 (60%) had shortness of breath, and 2 (40%) had fatigue. Therapies used for COVID-19 were antibiotics (four patients), glucocorticoids (four patients), antiviral therapy (three patients), immunomodulators (three patients), and mechanical ventilation (one patient). COVID-19 infection lasted for more than 30 days for three (60%) of the five patients, and one patient died of COVID-19.

**Table 1:** Characteristics of 5 patients with COVID-19 and colorectal cancer and 20 matched patients with COVID-19 without cancer.

Characteristics	No. of patients (%)		*P* value
	Colorectal cancer (*n* = 5)	Without cancer (*n* = 20)	
Sex			1
Female	2 (40)	8 (40)	
Male	3 (60)	12 (60)	
Age			1
>65 y	3 (60)	14 (70)	
≤65 y	2 (40)	6 (30)	
Comorbid conditions			
0	1 (20)	4 (20)	1
1	3 (60)	13 (65)	
2	0 (0)	0 (0)	
3	1 (20)	3 (15)	
Type of comorbid condition			
Diabetes	1 (20)	5 (25)	1
Hypertension	3 (60)	11 (55)	1
Coronary heart disease	1 (20)	2 (10)	0.504
Cerebrovascular disease	0 (0)	1 (5)	1
Renal disease	1 (20)	3 (15)	1
Liver disease	0 (0)	0 (0)	/
TNM disease stage			
I	2 (40)	/	
II	2 (40)	/	
III	1 (20)	/	
Tumor site			
Colon	1 (20)	/	
Rectum	4 (80)	/	
Laboratory findings			
White blood cell count, <4 × 10^9^/L	1 (20)	7 (35)	1
Lymphocyte count, <0.8 × 10^9^/L	4 (80)	4 (20)	0.023
Neutrophil count, <1.8 × 10^9^/L	1 (20)	1 (5)	0.367
Platelet count, <100 × 10^9^/L	0 (0)	1 (5)	1
Hemoglobin, <115 g/L	1 (20)	4 (20)	1
Hypersensitive C-reactive protein, >6 mg/L	5 (100)	6 (30)	0.009
Procalcitonin, >0.05 ng/mL	4 (80)	10 (50)	0.341
Lactate dehydrogenase, >300 U/L	3 (60)	3 (15)	0.070
D-dimer, >0.5 μg/mL	3 (60)	7 (35)	0.358
Alanine aminotransferase, > 40 U/L	0 (0)	2 (10)	1
Aspartate aminotransferase, >40 U/L	1 (20)	5 (25)	1
Total protein, <60 g/L	2 (40)	5 (25)	0.597
Albumin, <34 g/L	3 (60)	12 (60)	1
Globulin, <26 g/L	0 (0)	4 (20)	0.549
Creatinine, >120 μmol/L	1 (20)	4 (20)	1
Blood urea nitrogen, >8.8 mmol/L	1 (20)	3 (15)	1
CO_2_ CP, >29 mmol/L	1 (20)	3 (15)	1
Pulse rate, ≥90 bpm	2 (40)	7 (35)	1
Respiratory rate, >20 times/min	4 (80)	5 (25)	0.040
SpO_2_ (%), <93%	2 (40)	4 (20)	0.562
Signs and symptoms			
Body temperature, °C	5 (100)	17 (85)	1
Cough	4 (80)	16 (80)	1
Sputum	1 (20)	6 (30)	1
Hemoptysis	0 (0)	1 (5)	1
Shortness of breath	3 (60)	12 (60)	1
Diarrhea	0 (0)	1 (5)	1
Nausea or vomiting	0 (0)	1 (5)	1
Fatigue	3 (60)	7 (35)	0.358
Myalgia	0 (0)	1 (5)	1
Anorexia	0 (0)	1 (5)	1
Sore throat	1 (20)	0 (0)	0.200
CT findings (bilateral)	5 (100)	18 (90)	1
Treatments			
Antibiotics (yes)	4 (80)	18 (90)	0.504
Antiviral therapy (yes)	3 (60)	7 (35)	0.358
Immunomodulators (yes)	3 (60)	8 (40)	0.623
Systemic glucocorticoids (yes)	4 (80)	8 (40)	0.160
Mechanical ventilation	1 (20)	1 (5)	0.367
COVID-19 > 30 days	3 (60)	7 (35)	0.358
Treatment in intensive care unit	1 (20)	1 (5)	0.367
Clinical outcome (death)	1 (20)	1 (5)	0.367

Next, we compared these results with findings from 20 patients with COVID-19 but without cancer who had been propensity-score matched in a 1:4 ratio on the basis of age, sex, and comorbid conditions. The two groups were well balanced, with no significant differences between them. However, cancer patients experienced significantly higher rates of lymphopenia (80% vs. 20%, *P* = 0.023), high hypersensitive C-reactive protein levels (100% vs. 30%, *P* = 0.009) and high respiratory rates (>20/min) (80% vs. 25%, *P* = 0.040). Apparent (though non-significant) differences between cancer patients and non-cancer controls included having high lactate dehydrogenase levels (60% vs. 15%, *P* = 0.070); receipt of antiviral therapy (60% vs. 35%, *P* = 0.358), glucocorticoids (80% vs. 40%, *P* = 0.160), and mechanical ventilation (20% vs. 5%, *P* = 0.367); having COVID-19 for longer than 30 days (60% vs. 35%, *P* = 0.358); and having a higher death rate (20% vs. 5%, *P* = 0.367) (Table 1).

We systematically investigated the expression of ACE2 and TMPRSS2 in human tumor and normal colorectal tissues by using both bulk and single-cell RNA-sequencing datasets, and found both receptors to be highly expressed in colorectal epithelial cells. We further found that patients with colorectal cancer and COVID-19 were more likely to have lymphopenia and higher respiratory rates and hypersensitive C-reactive protein levels than were patients with COVID-19 but without cancer. These results suggest that patients with colorectal cancer may be particularly vulnerable to infection with SARS-CoV-2 and thus extra precautions should be taken to prevent them from developing COVID-19.

## Methods

### Study design and participants

The 5 COVID-19 patients with colorectal cancer and the 20 patients with COVID-19 without cancer were identified from 135 patients admitted to Wuhan Hankou Hospital from 11 January 2020 to 12 February 2020. Demographic, clinical, and lab data were obtained from the medical record system. COVID-19 diagnosis was confirmed by real-time polymerase chain reaction tests for SARS-CoV-2. This study was approved by the Ethical Committee of Wuhan Hankou Hospital (HKYY-2020-028), with a waiver of written informed consent.

We performed 1:4 propensity score matching (PSM) to select 20 matched COVID-19 patients without cancer to compare with the five COVID-19 patients with colorectal cancer. PSM was done based on age, sex, and comorbid conditions recognized as being potential risk factors for COVID-19 prognosis^21–23^ and randomly sorted by using the nearest neighbor technique with acceptable distance (with a caliper of 0.02 to obtain robust comparison results) of propensity scores.

### Analysis of single-cell and bulk RNA expression matrices

Expression of ACE2 and TMPRSS2 in tumor and adjacent normal tissue samples of human lung, esophagus, stomach, liver, colon, and rectum was analyzed by bulk RNA sequencing of samples from The Cancer Genome Atlas via the University of California Santa Clara’s Xena website (https://xenabrownser.net). Single-cell RNA expression matrices for human epithelial and non-epithelial cells from normal and cancerous colorectal tissues were downloaded from the Gene Expression Omnibus (GEO, numbers GSE81861 and GSE146771)^24,25^. After quality-control processing of the single-cell RNA expression data, and we selected eligible cells for downstream analysis, which was done with the Seurat package^26^ and included identification of highly variable genes, unsupervised graph-based clustering, differentially expressed genes, and dimension reduction using principal component analysis and uniform manifold approximation and projection analysis. We further analyzed the correlation of ACE2 and TMPRSS2 with enterocyte markers with Pearson correlation coefficients.

## Supplementary information

nr-reporting-summary

## Data Availability

The data generated and/or analysed during this study are described in the figshare metadata record: 10.6084/m9.figshare.13265096^27^. The expression profiling data underlying Figs. 2–4 are openly available at the Gene Expression Omnibus repository at the following two series accessions: https://identifiers.org/geo:GSE81861^28^ and https://identifiers.org/geo:GSE146771^29^. The COVID-19 patient data are stored in the file COVID-19 patient data.xlsx. This file is not publicly available to protect patient privacy. Contact Prof. Qinyong Hu (rm001223@whu.edu.cn) with data requests. The following samples from The Cancer Genome Atlas were analysed by bulk RNA sequencing and are openly available via the University of California Santa Clara’s Xena website (https://xenabrownser.net): TCGA-LUAD.htseq_fpkm.tsv, TCGA-LUSC.htseq_fpkm.tsv, TCGA-ESCA.htseq_fpkm.tsv, TCGA-COAD.htseq_fpkm.tsv, TCGA-STAD.htseq_fpkm.tsv, TCGA-LIHC.htseq_fpkm.tsv, TCGA-READ.htseq_fpkm.tsv. These data underlie Fig. 1 in the related manuscript.

## Code availability

The code that supports the findings of this study are available from the corresponding author on reasonable request.
